# Toxicity of Single and Mixed Contaminants in Seawater Measured with Acute Toxicity Bioassays

**DOI:** 10.1100/tsw.2002.221

**Published:** 2002-04-25

**Authors:** A.R. Fernandez-Alba, L. Piedra, M. Mezcua, M.D. Hernando

**Affiliations:** Pesticide Residue Research Group, Faculty of Sciences, University of Almería, 04120, Almería, Spain

**Keywords:** biocides, methyl tertiary-butyl ether (MTBE), acute toxicity, mixtures, Vibrio fischeri, Daphnia magna

## Abstract

Different types of organic pollutants commonly detected in seawater have been evaluated by acute toxicity bioassays. Vibrio fischeri, Daphnia magna, and Selenastrum capricornotum were selected to test toxic effects of individual compounds and mixtures of these compounds, obtaining EC_50_ values in the range of 0.001 to 28.9 mg/l. In the case of mixtures, synergistic toxic responses were seen for a clear majority of the cases (>60%). Mixtures containing methyl-tertiary-butyl ether (MTBE) exhibit accelerated processes that result in a change in concentration required to produce a toxic effect; for example, in the case of mixtures containing MTBE and Diuron and Dichlofluanid.

### INTRODUCTION

Hundreds of new chemicals are introduced into commerce every year and may eventually end up in the environment. Many of these new and existing compounds are toxic to the biosphere. Of special concern are compounds that, because of the nature of their application in agricultural, industrial, and tertiary activities, are deliberately released into the environment in large amounts. Different types of organic pollutants such as pesticides are detected as consequence of their ubiquitous use in groundwater, surface water, and seawater[1,2].

Pesticides used as antifouling used as antifouling agents are toxic compounds and are commonly encountered as mixtures with similar or other types of chemicals in aquatic ecosystems. Other contaminants include the fuel oxygenate methyl tertiary-butyl ether (MTBE), which is widely used as an additive in gasoline; its ubiquitous use results in its detectable presence in these systems[3,4]. These mixtures of compounds have been detected in the Mediterranean Sea at concentrations up to 0.9 and 0.8 mg/l for Irgarol and Diuron, respectively[1,2]. They may pose bigger problems because the toxicity of a mixture is not easily linked to individual toxicities of components in the mixture; synergistic and antagonistic responses are known to occur[5,6,7,8]. A broader knowledge, therefore, of the ecotoxicity of the single compound and of mixtures of organic pollutants and their persistence is a matter of extreme public concern.

Traditionally, chemical characterization is performed to evaluate pollution episodes in water samples, but the ecotoxicological impact is not fully determined. In this sense, bioassays permit the elucidation of the acute toxicity of a water sample and represent a very interesting and complementary evaluation. In this work, three different types of bioassays have been optimized and tested to evaluate the toxic impact of several types of organic pollutants in water.

### EXPERIMENTAL METHODS

#### Test Chemicals

The following contaminants were tested: Carbofuran, Formetanate, Irgarol 1051 (2-methylthio-4-tert-butylamino-6-cyclopropylamino-s-triazine), Kathon 5287 (4,5-dichloro-2-(n-octyl)-3(2H)-isothiazolone), Dichlofluanid (N-dimethyl-N-phenylsulphamide), Diuron (3-(3,4-dichlorophenyl)-1,1-dimethylurea), 2-thiocyanomethylthiobenzothiazole (TCMTB), and MTBE. All chemicals selected were purchased in the highest purity available from Ciba-Geigy (Barcelona, Spain), Rohm and Hass (Philadelphia, PA, U.S.A.), Chem Service, Inc. (West Chester, PA), and Riedel-de Haën (Seelze, Germany).

Individual and mixture stock solutions were prepared in distilled water for the *Vibrio fischeri* assay and in culturing medium for the *Daphnia magna* and *Selenastrum capricornotum* bioassays. For the *V. fischeri* assay, the osmolality of solutions was adjusted to 2% NaCl for optimal performance. Mixtures containing MTBE were prepared at a concentration of 100 mg/l and the effective concentration level ($EC_{50}$) of each individual pesticide.

#### Toxicity Bioassays

#### Vibrio fischeri *Bioluminescence Assay*

The effect of test compounds on the luminescent bacterium *V. fischeri* was evaluated using the Biotox™ test with 30-min $EC_{50}$ (Bio-Orbit Oy, Turku, Finland). The $EC_{50}$ was determined as the concentration of a test sample that caused a 50% reduction in light output. The acute bioluminescence assay was carried out according to International Organization for Standardization (ISO) 11348[9].

#### Daphnia magna *Motility Assay*

The toxicity of the compounds to the crustacean species (*D. magna*) was assessed using a commercially available Toxkit, Daphtoxkit™ (Creasel Ltd., Deinze, Belgium). The toxicity studies were performed in accordance with testing conditions prescribed by Organization for Economic Cooperation and Development (OECD) Guideline 202[10]. The $EC_{50}$ was determined as the concentration estimated to immobilize 50% of the daphnids after 24 and 96 h of exposure.

### *Selenastrum capricornotum* Growth Inhibition Assay

The algal test was performed in accordance with the prescriptions of the OECD (Guideline 201)[11] and using the commercially available Toxkit, Algaltoxkit™ (Creasel Ltd., Deinze, Belgium). Inhibition of the algal growth relative to control was determined by measurement of the OD in a spectrophotometer with a filter at 670 nm. The 72-h $EC_{50}$ value in *S. capricornotum* was calculated as the concentration of test substance which results in a 50% reduction in growth relative to the control.

## RESULTS AND DISCUSSION

### Single Substance Toxicity of Biocides

Multiple biosensors are required to uncover the many potential biochemical interactions of a toxicant in any life form. This study presents a detailed analytical assessment of several bioassays for different types of contaminants for acute toxicity (Tables 1 and 2); these bioassays include bacterial, invertebrate, and algal biochemistry. Three rapid and relatively inexpensive toxicity bioassays were used: (i) the luminescent bacterium *V. fischeri* (Biotox™); (ii) the aquatic invertebrate *D. magna* (Daphtoxkit™)[10]; and (iii) the microalgae *S. capricornotum* (Algaltoxkit™)[11]. These three tests

**TABLE 1**
**Linear Range for Single Pesticides Analyzed by Biotox™, Algaltoxkit™, and Daphtoxkit™**

| | Linear Range ($mg\ l^{-1}$) | | |
|---|---|---|---|
| **Pesticides** | **Biotox™** | **Algaltoxkit™** | **Daphtoxkit™** |
| Formetanat | 1.6–25.6 | — | 0.1–1 |
| Carbofuran | 4.4–70 | — | 0.02–0.2 |
| Kathon 5287 | 0.002–0.014 | 0.001–0.004 | 0.005–0.014 |
| Irgarol 1051 | 5–10 | 0.005–0.02 | 1–10 |
| Diuron | — | 0.025–0.1 | 1–10.8 |
| Dichlofluanid | 0.023–0.092 | 0.066–0.26 | 0.5–2 |
| TCMTB | 0.023–0.092 | 0.21–0.84 | 0.01–0.1 |

**TABLE 2**
**$EC_{50}$ Values for Single Pesticides Analyzed by Biotox™, Algaltoxkit™, and Daphtoxkit™**

| | $EC_{50}$ ($mg\ l^{-1}$) | | |
|---|---|---|---|
| **Pesticides** | **Biotox™ Exposure (min) 30** | **Algaltoxkit™ Exposure (h) 72** | **Daphtoxkit™ Exposure (h) 48** |
| Formetanat | 4.8 ± 0.7 | — | 0.07 ± 0.005 |
| Carbofuran | 28.9 ± 2.5 | 7.6 ± 1.3 | 0.02 ± 0.007 |
| Kathon 5287 | 0.003 ± 0.0003 | 0.003 ± 0.0003 | 0.001 ± 0.0007 |
| Irgarol 1051 | 15.5 ± 2.7 | 0.01 ± 0.001 | 7.3 ± 1.24 |
| Diuron | — | 0.045 ± 0.004 | 8.6 ± 1.3 |
| Dichlofluanid | 0.06 ± 0.0118 | 0.13 ± 0.02 | 1.05 ± 1.33 |
| TCMTB | 0.039 ± 0.0078 | 0.43 ± 0.03 | 0.046 ± 0.005 |

were optimized to detect pesticides over a concentration range of ppb levels that permit their use as screening method (Table 1).

The toxicity ($EC_{50}$) for each bioassay was determined as the concentration of test sample that causes a 50% reduction in the biological response (Table 2). Acute toxicity was evaluated on water samples spiked with various amounts of the following pesticides: Carbofuran, Formetanate, Kathon 5287 (Sea nine 211), Irgarol 1051, Diuron, Dichlofluanid, and TCMTB. Some of these compounds also are commonly used in agricultural activities. Three toxicological tests were optimized to detect pesticides over a concentration range of ppb levels, which permit their use for purposes of toxicological screening of environmental samples.

### Mixture Toxicity of Biocides

So far, toxicity of compounds has been assessed using single compounds because of a lack of standardized methods for assessing synergistic toxicity[6,12]. Nevertheless, the existence of synergistic effects is well known[6,10,12], and there is a clear need to quantify the aggregate toxic impact of pesticide mixtures. For example, a recent report by the U.S. Geological Survey on the quality of the aquatic environment emphasized the need to consider synergistic effects of chemical mixtures on aquatic toxicity[13].

The methods used to relate the individual component toxicity to the expected toxicity of the mixture have been discussed in the literature[6,14]. Concentration addition has gained some acceptance as having a reasonable pharmacological basis. According to this model of a mixture's toxicity, the toxicities of individual components are additive if the substances act on common active sites of metabolic enzymes and if a constituent chemical acts as a solvent for the other components[6,10,12]. In this situation, the toxic effect of any component can be replaced with an equieffective amount of another chemical. Following this model, the concentration of each toxicant is expressed as a fraction of its $EC_{50}$ (toxic unit, TU). In the case of concentration addition, the 50% response of a mixture of chemicals is obtained when the sum of TUs ($\Sigma$ TU) of all constituents equals unity. In contrast, when $\Sigma$ TU exceeds unity, the combined effect of toxicants is more than additive (i.e., a synergistic response). Similarly, when $\Sigma$ TU is less than unity, then the toxic response is less than additive; i.e., the toxicants act antagonistically.

Based on experimental $EC_{50}$ values of constituent components, the expected toxicities of the mixtures can be calculated by adding the concentrations. A comparison of the calculated toxicity with the experimentally observed toxicity of the mixture determines if the toxic response is additive, synergistic, or antagonistic. Table 3 documents the observed effects for binary mixtures.

**TABLE 3**
**Synergistic Effects for Binary Mixtures of Pesticides Analyzed by Biotox™, AlgaltoxKit™, and Daphtoxkit™**

| Mixtures | Biotox™ | Algaltoxkit™ | Daphtoxkit™ |
|---|---|---|---|
| Irgarol 1051-Diuron | Synergistic | Synergistic | Synergistic |
| Irgarol 1051-Kathon 5287 | Antagonistic | Additive | Additive |
| Irgarol 1051-Chlorothalonil | Synergistic | Synergistic | Antagonistic |
| Irgarol 1051-Dichlofluanid | Additive | Additive | Synergistic |
| Irgarol 1051-TCMTB | Synergistic | Synergistic | Synergistic |
| Formetanate-Carbofuran | Additive | — | Additive |

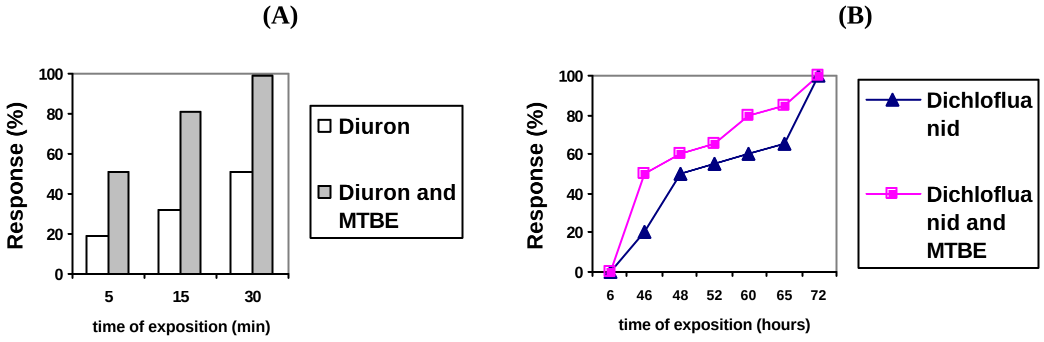

**FIGURE 1.** Increase in the toxic effect by the presence of MTBE in combination with Diuron and Dichlofluanid. (A) Analysis by *V. fischeri* toxicity assay. (B) Analysis by *D. magna* toxicity assay.

### Mixture Toxicity of Biocides and MTBE

It is of interest to consider the related effects on toxicity of additional compounds that have no apparent toxic effects but can be present in aquatic systems in combination with pesticides. This is the case with MTBE, a ubiquitous additive of gasoline. As it is evaluated, this chemical presents very low toxic effects on aquatic organisms alone[3]; however, if we take into consideration different mixtures of pesticides and MTBE, important effects can be observed on some aquatic organisms such as *D. magna* or *V. fischeri*. Mixtures containing MTBE at levels of concentration of 100 mg/l and $EC_{50}$ of each individual pesticide were evaluated. This is shown in Fig. 1 where the presence of MTBE in combination with Dichlofluanid or Diuron shows an important increase in the toxic effect, resulting in a 50% increase in response with Diuron on *V. fischeri* and a 50% increase in response ($EC_{50}$) in 5 min while 30 min are required to obtain the same for the single pesticide. In the case of the mixture of MTBE and Dichlofluanid, the toxic effect on *D. magna* increased by 30% and the $EC_{50}$ response occurred 2 h faster than the response to Dichlofluanid alone. The increase in the magnitude of the toxic effect and the decrease in the time required to manifest toxic effects might be associated with alterations in the toxicokinetics phase where processes of uptake through cell act as active processes.

### CONCLUSIONS

Toxicity testing very adequately complements other chemical analysis and has a clear role in the evaluation of water quality, but a considered selection of testing methods is essential for obtaining results that are relevant in establishing water quality.

A synergistic toxic response was seen for a clear majority of the cases, suggesting that a consideration of individual component toxicity alone is not sufficient for determining the environmental impacts of toxicants.

Interactions in mixtures can generate accelerated processes of uptake of contaminants in the organisms and can arise before the effective concentration that causes the toxic effect, as in the cases of mixtures MTBE with Diuron and Dichlofluanid.
